# Perioperative management of upper tract urothelial carcinoma in the Nordic countries

**DOI:** 10.1186/s12894-024-01515-7

**Published:** 2024-06-25

**Authors:** Kimie Oedorf, Erik Skaaheim Haug, Fredrik Liedberg, Riikka Järvinen, Sigurdur Gudjonsson, Peter J. Boström, Tomas Jerlström, Gigja Gudbrandsdottir, Jørgen Bjerggaard Jensen, Gitte Wrist Lam

**Affiliations:** 1 Department of Urology, Herlev and Gentofte Hospital, Copenhagen, Denmark; 2 Department of Urology, Vestfold Hospital Trust, Toensberg, Oslo Norway; 3 Department of Urology, Skaanes Universitetssjukhus, Malmö, Sweden; 4 Department of Urology, University of Helsinki and Helsinki University Hospital, Helsinki, Finland; 5 Faculty of Medicine, Department of Urology, University of Iceland, Landspitali University Hospital, Reykjavik, Iceland; 6 Department of Urology, Turku University Hospital, University of Turku, Turku, Finland; 7 Department of Urology, School of Medical Sciences, Faculty of Medicine and Health, Örebro University, Örebro, Sweden; 8 Department of urology, Haukeland University Hospital, Bergen, Norway; 9 Department of Urology, Aarhus University Hospital, Aarhus, Denmark

**Keywords:** Upper tract urothelial carcinoma, Nephroureterectomy, Survey, Guideline

## Abstract

**Background:**

Upper tract urothelial carcinoma (UTUC) is a rare malignancy, with typically only few new cases annually per urological department. Adherence to European association of urology (EAU) guidelines on UTUC in the Nordic countries is unknown. The objective of this survey was to examine the implementation of EAU guidelines, the perioperative management and organization of the treatment of UTUC in the Nordic countries.

**Methods:**

The electronic survey was distributed to 93 hospitals in the Nordic countries performing radical nephroureterectomy (NU). The survey consisted of 57 main questions and data was collected between December 1st, 2021 and April 23rd, 2022.

**Results:**

Overall response rate was 47/93 (67%) with a completion rate of 98%. Five out of the 6 examined subjects on diagnostic practice are applied by ≥ 72% of the participating centers. NU as treatment for high-risk UTUC is performed by 37/47 (79%), and 91% include a bladder cuff excision.

**Conclusions:**

Adherence to EAU guidelines is high on diagnostic practice in the Nordic countries, whereas disease management is less coherent.

**Supplementary Information:**

The online version contains supplementary material available at 10.1186/s12894-024-01515-7.

## Introduction

Upper tract urothelial carcinoma (UTUC) is a relatively rare disease, accounting for 5–10% of all urothelial cancers with an estimated annual incidence of 1–2 cases per 100,000. The rate of metastatic disease at presentation is 7–9% [1, 2].

Studies on temporal trends reveals high proportion of locally advanced or metastatic disease (60%) and high-grade (70%) tumor, with a shift towards more aggressive disease over the last two decades [3].

Gold standard treatment for non-metastatic high-risk UTUC is radical nephroureterectomy (NU) with bladder cuff excision. In patients with low-risk tumors, kidney-sparing endoscopically treatment (or segmental ureterectomy) is recommended [2]. However, it is impossible to determine T-stage before histology after NU is available. The imaging modalities are suboptimal, and pathological evaluation of a biopsy will often be restricted to only evaluating grade of the tumor, not stage. Therefore, making decision onf type of surgical treatment, whether to perform lymphadenectomy (LND) and selection of patients for systemic chemotherapy preoperatively is difficult. To assist in this process, guidelines recommend predictive tools based on preoperative patient and tumor characteristics when selecting treatment modality [2]. However, it is known from prior studies that there are discrepancies between guideline recommendations and daily practice in the management of urothelial cancer of the bladder [4–7]. To the best of our knowledge, adherence to the EAU guidelines on UTUC in the Nordic countries is unknown.

The objective of this survey was to study the implementation of EAU guidelines, the perioperative management and organization of the treatment of UTUC and to examine if hospital volume is associated with practice patterns in the Nordic countries: Norway, Sweden, Finland, Iceland and Denmark.

## Materials and methods

The study was performed as a multicenter survey including 93 centers in Norway, Sweden, Finland, Iceland, and Denmark. The members of the 5 countries represented in Nordic urothelial cancer group (NUCG) identified the hospitals and centers that were included in the study, i.e. centers believed to perform nephroureterectomy.

One surgeon from each of the 93 centers was asked to complete a survey dispensed electronically via Survey Monkey^®^ between December 1, 2021 – April 23, 2022. Reminders were sent through Survey Monkey^®^ and by the members of NUCG during this period. The questionnaire consisted of 57 multiple choice or open questions and 13 elaborative questions as seen in supplemental material 1. Questions addressed the preoperative and diagnostic practice, surgical volume of radical nephroureterectomy for UTUC, surgical technique, organization and team-structure, number of surgeons performing the procedure, use of chemotherapy, multidisciplinary conferences, practice for intraoperative management and postoperative follow-up. Participants were given the opportunity to not respond if the answer was unknown or if a main question was not relevant. Data were collected from returned questionnaires and is presented descriptively. Low volume centers were defined as centers who perform < 10 NU/year and high volume centers > 10 NU/year. To compare categorical variables between low- and high-volume centers, chi-square test was used. P-value < 0.05 is considered to indicate statistical significance.

## Results

Returned questionnaires were received from 70 out of the 93 invited centers, out of these 8 were duplicate responses from the same institution. Thus, the overall response rate for the survey was 67% (62/93). As seen in the in-/exclusion flowchart of participants in supplemental material 2, 47 returned questionaries were used for data analyses. Iceland, Denmark and Finland all had a response rate of 100%. The completion rate for the 57 main questions was 98% (56/57).

### Demography and volume

The catchment population for UTUC of included centers is shown in supplemental material 3. Number of NU performed annually is < 10 in 58% (27/47), 11–50 in 36% (17/47) and 51–75 in 6% (3/47) of the included centers. Cystectomy is performed at overall 53% (25/47) of centers, and by 37% (10/27) of low volume centers and 71% (15/20) of high volume centers.

The number of surgeons who perform NU in the participating centers is shown in Table 1. All centers with 4–5 different surgeons performing NU are in the < 50 NU/year-group, and all centers that perform 50–75 NU/year have 2–3 different surgeons.

**Table 1:**
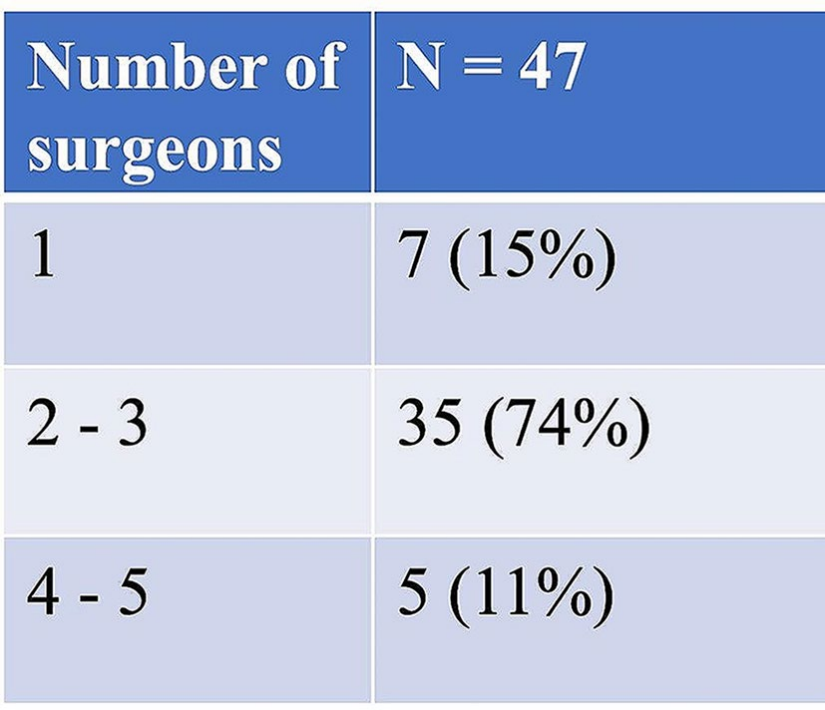
Number of surgeons performing NU in the department

All but 6% (3/47) of the surveyed centers say that they have Multidisciplinary conferences on UTUC, and the different specialties that attend the conferences and by which proportion, is shown in supplemental material 4.

**Fig. 1:**
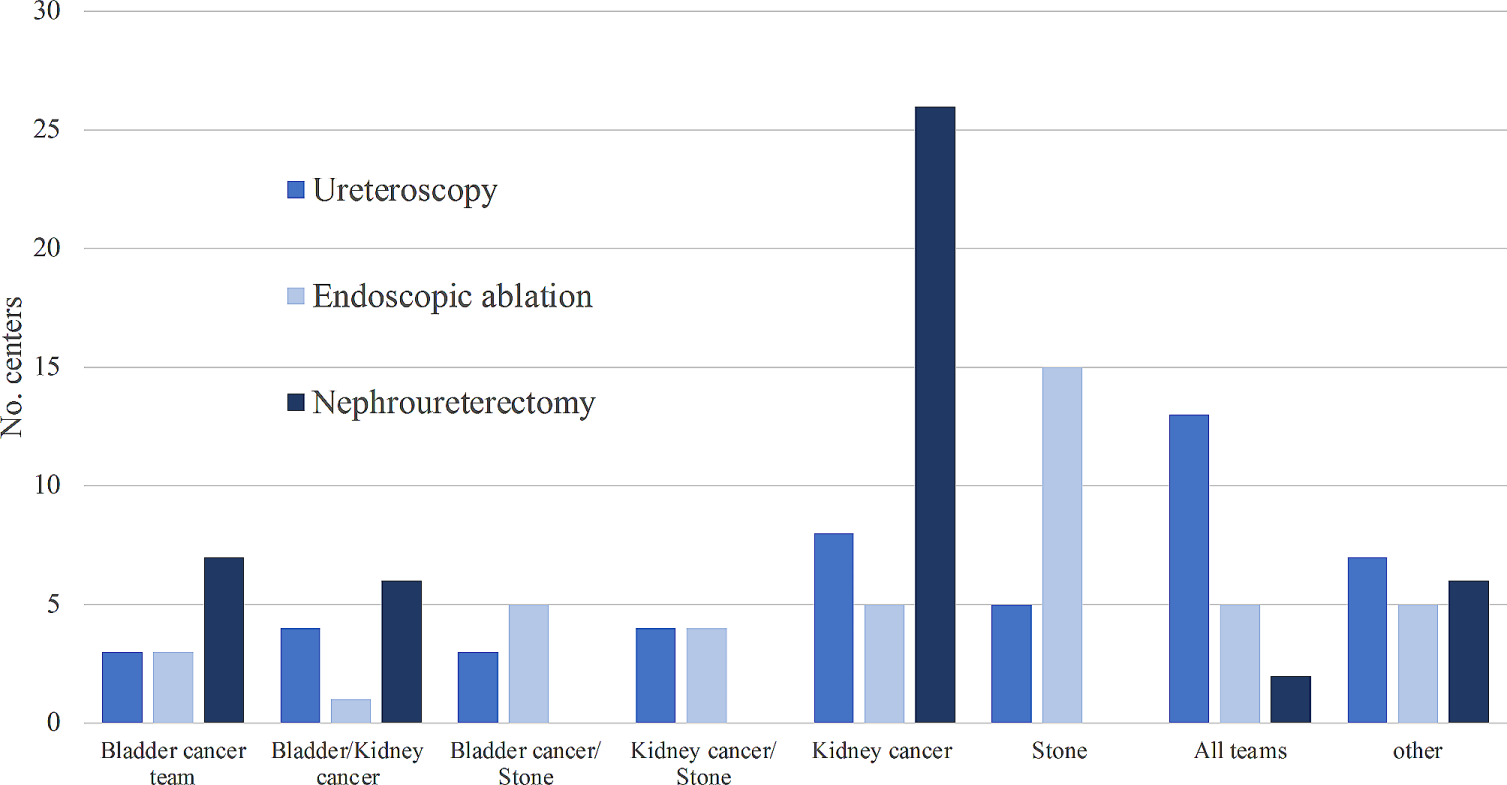
Organization by subspecialty of treatment

The organization by subspecialty of diagnostic, endoscopic procedures and radical treatment is shown in Fig. 1.

19% (9/47) of the responding centers do not have an internal subspecialization within the urological department. One out of two, 45% (20/47), of responders have a specific team that perform both diagnostic ureteroscopy and endoscopic ablation.

Surgeons in the kidney cancer team most frequently carry out the NU in 55% (26/47) of the centers. Four centers refer patients for endoscopic treatment. Around two-thirds (37/47) of participants register data on patients treated surgically for UTUC, mostly in retrospective databases.

### Guidelines - Diagnostic practice

**Fig. 2:**
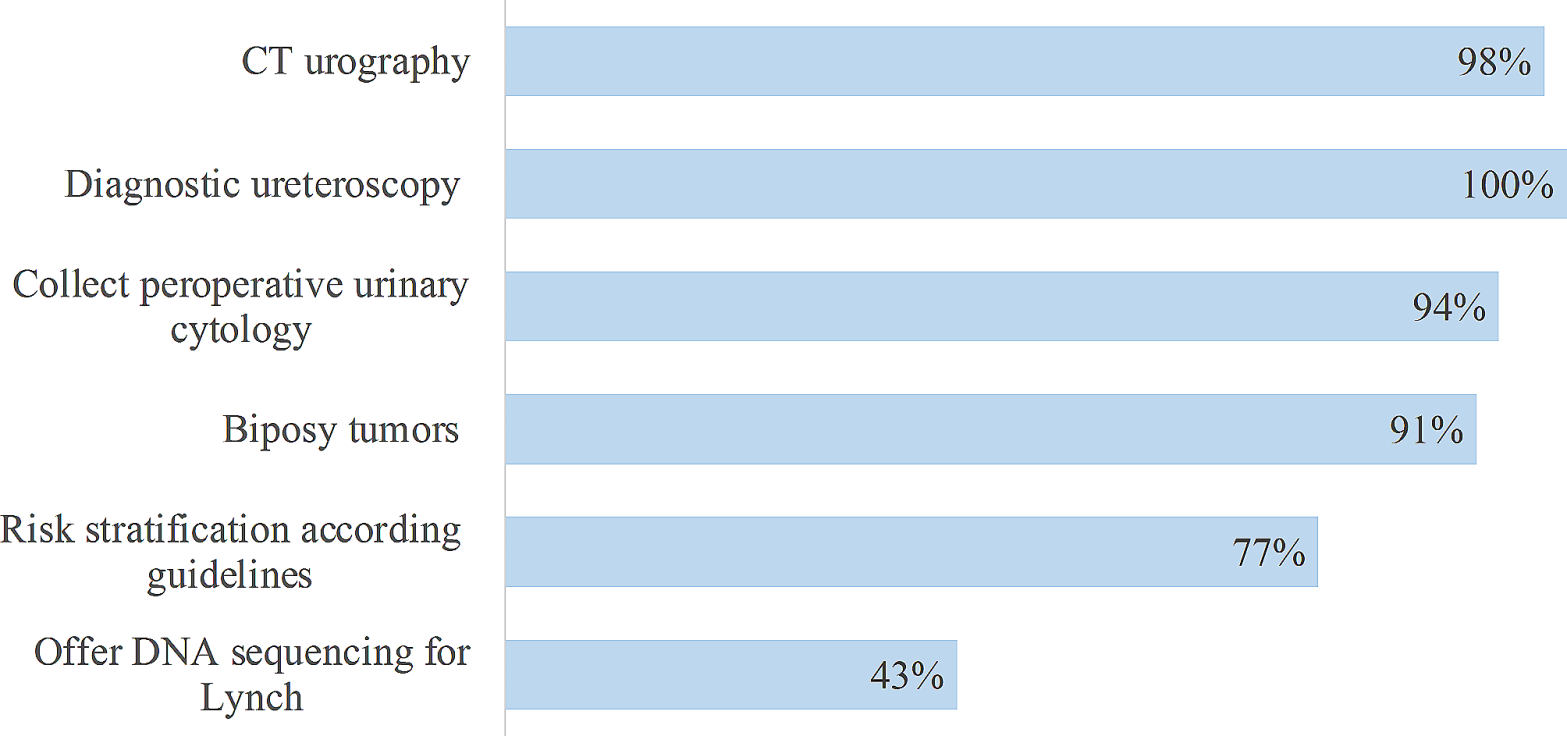
Adherence to EAU guidelines on diagnostic

Results on the surveyed elements recommended by EAU concerning diagnostic practice is shown in Fig. 2.

As primary routine examination for TNM classification, CT-urography is used by 98% (46/47) of responders, and 46% (22/47) in combination with PET-CT-scan.

15% (7/47) use all tree modalities: CT-urography, PET-CT and/or MR-urography in selected cases. Preoperative renography is done at 36% (17/47) of centers, either always or in most cases. All centers perform diagnostic ureteroscopy, 77% (36/47) if imaging and cytology is not sufficient for diagnosis, and 23% (11/47) in all cases. Cytology is collected from renal cavities during endoscopy in all cases by 74% (35/47) and when diagnosis is unclear by 19% (9/47) of centers. Figure 2 shows that 91% (43/47) take biopsies endoscopically before treatment. Out of these, 15 responded always, 19 “In most cases” and 9 “In few selected cases”. The EAU recommendation to use preoperative risk-stratification is followed by 77% (36/47). The remaining centers stratify according to TNM-stage. Routine DNA sequencing for patients at high-risk for Lynch syndrome is offered by 43% (20/47) of the centers and 70% (33/47) has formal follow-up programs for patients with Lynch syndrome.

### Guidelines - disease management

**Fig. 3:**
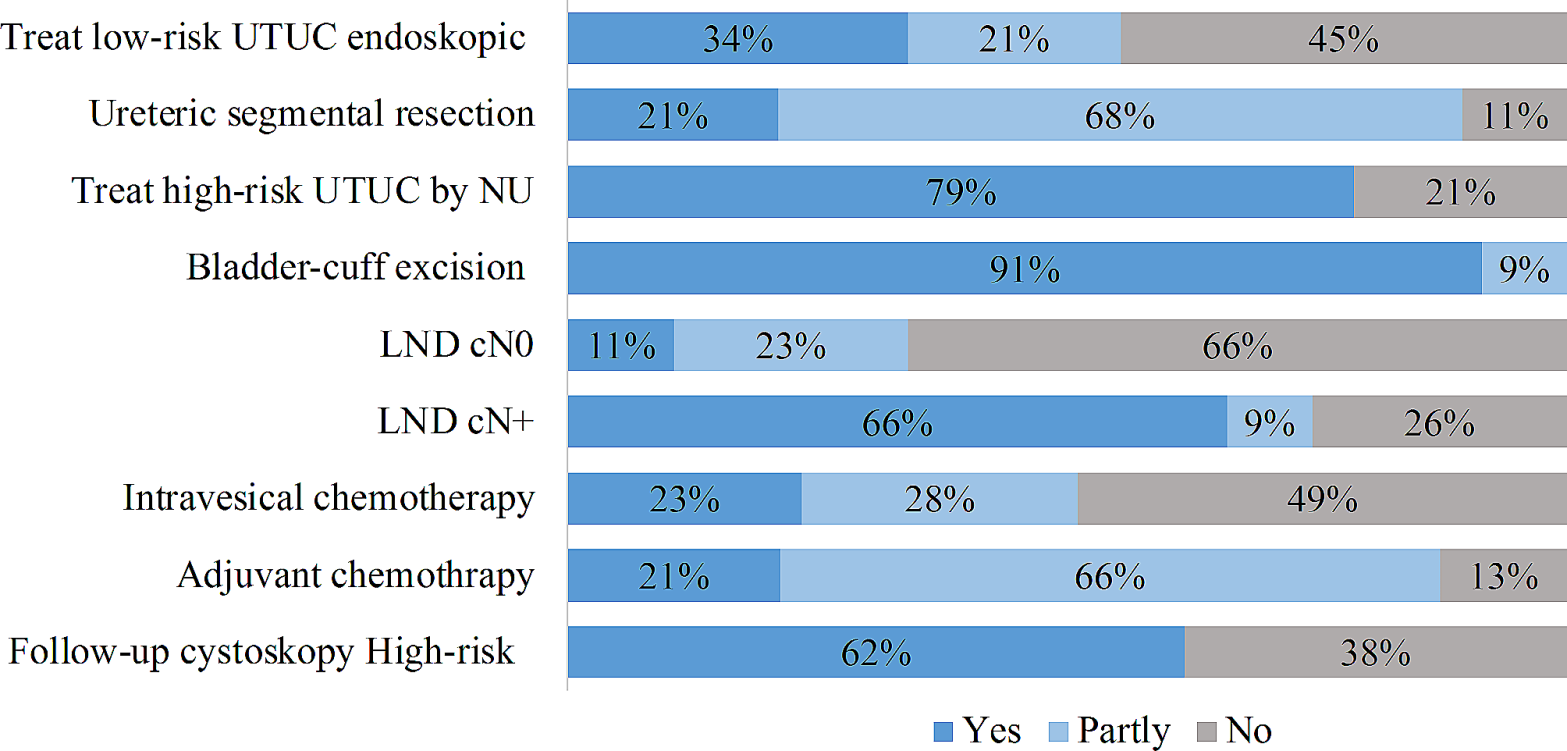
Adherence to EAU guidelines on treatment

The surveyed parameters recommended by EAU concerning disease management and follow-up is presented in Fig. 3.

Endoscopic treatment is performed by 91%, however only 34% (16/47) select cases for endoscopic treatment as recommended by EAU. The remaining 21% that follow guidelines partly, select patients for endoscopic treatment by criteria that consists of some of the elements in the guidelines. Majority use the need for preservation of kidney function and not “low-risk tumors” as selection criteria. Likewise, the criteria for selection of patients treated by segmental ureteric resection vary. Majority of centers do either not follow EAU guidelines (21% (8/38)) or only partly (68% (26/38)).

When method of NU is addressed, 62% (29/47) use robot-assisted laparoscopic approach. Out of these, 4 participants did not answer by which percentage this method is used. For the remaining 73% (19/26), robot-assisted NU is used in ≥ 80% of procedures. Only 11% (5/47) carry out template-based lymph (LND) node dissection concomitant to NU in all cases of cN0, but 23% (11/47) do so in selected cases, most in suspicion of locally advanced tumor. When the same question was asked for cN+, the proportion of participants that perform LND in all cases rise to 66% (31/47) and 74% (35/47) when including those that do so in selected cases.

Around half (57%, 27/47) of participating centers instill topical agents in the upper urinary tract in selected cases, mostly in case of CIS and in case-by-case evaluation.

Around half of participating centers administer intravesical chemotherapy postoperatively after NU, 23% (11/47) always and 28% (13/47) in selected cases. In all but one of the centers that follow this recommendation, the administration is done before day 10 postoperatively.

Adjuvant chemotherapy after NU is used in all cases by 21% (10/47) and in selected cases by 66% (31/47) of the centers. These percentages change to 10% (5/47) all cases and 45% (21/47) for selected cases when concerning neo-adjuvant chemotherapy.

### Guidelines - follow-up

After NU for low-risk tumors, 79% (37/47) perform cystoscopy at 3, 9 and 12 months, then yearly for 5 years. 10% (5/47) do cystoscopy by a different frequency the first year, then yearly until 5 years. The remaining 10% (5/47) have other local regimens for follow-up. Urography is done in all cases as follow up after NU for low-risk tumors by 32% (15/47) of participants, and by 25% (12/47) in selected cases.

### Outcome and surgical volume

**Fig. 4:**
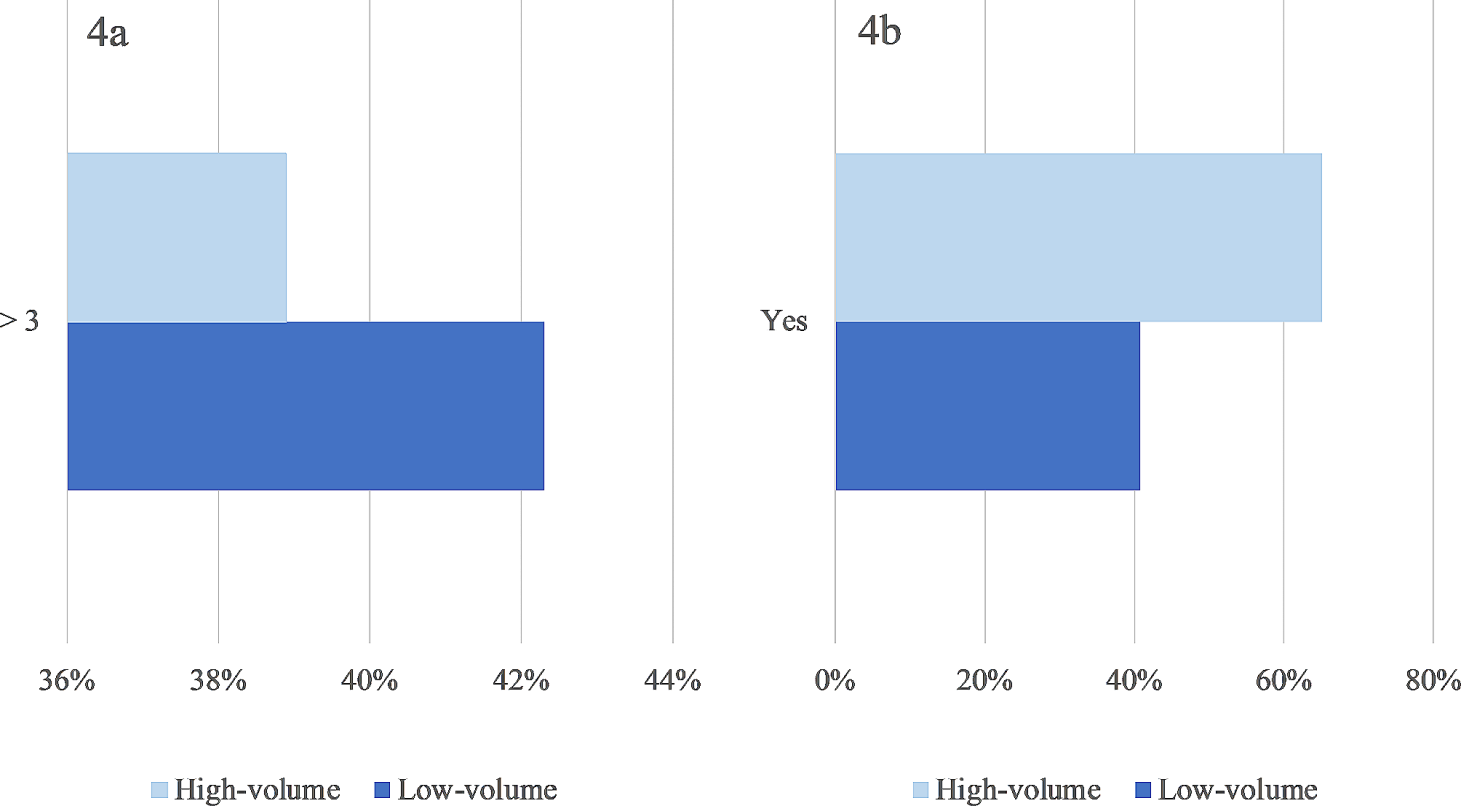
**a**: Difference in length of stay between high- and low-volume centers. **b**: Usage of intravesical chemotherapy in high- and low-volume centers

Three of the responding centers did not know the estimated length of stay (LOS), leaving 44 for outcome-analysis: 59% (26/44) of the centers that perform < 10 NU/year (low volume) and 41% (18/26) of the centers that perform 10–75 NU/year. As seen in Fig. 4a LOS after NU is < 3 days in 59% (26/44) of participating centers, the remaining reported LOS as 3 days or more.

The same figure shows that there is no difference in LOS between low- and high-volume centers. A chi-square test of independence confirms no significant association between surgical volume and LOS. *p*-value: 0.82.

Figure 4b shows the usage of postoperative intravesical chemotherapy stratified by surgical volume per center. A majority of low volume centers seems represented in the group of participants, that do not administer postoperative intravesical chemotherapy. However, no statical association is seen between surgical volume and usage of intravesical chemotherapy. *p*-value: 0.099.

## Discussion

The present study shows similar perioperative routines in Nordic centers performing NU among the majority of the examined subjects on the treatment of UTUC.

The extent of adherence to EAU guidelines concerning diagnostic practice was high. Five out of 6 examined components recommended by EAU as a part of the diagnostic practice, were adopted by ≥ 72% of the participating centers. This differs from other studies on implementation of guidelines on disease management in European countries [4, 5, 7] and other international studies [8, 9]. An online survey proposed to physicians in the field of bladder cancer from nine European Countries found that up to 45% of high-risk disease did not receive a re-TURB and adjuvant instillation. This despite the fact that 87% of participants declare to follow EAU guidelines [4]. On the other hand, the current findings are similar to a prior survey in the Nordic countries for the general management of radical cystectomy and adherence to enhanced recovery protocols and EAU guidelines [10].

The EAU guidelines recommend that patients identified as being at risk of for Lynch syndrome should undergo DNA sequencing and family counselling, but there is no specified follow-up program for UTUC fore these patients in the guidelines.

Under half of the participating centers follow EAU guidelines concerning DNA sequencing for patients that are highly suspected of having Lynch syndrome. This survey did not address whether participants use the Amsterdam criteria when screening for Lynch syndrome, or what reason participants have for not following guidelines. National guidelines vary on the subject of offering DNA sequencing for Lynch syndrome [5, 11], which might be a part of the explanation for the lack of implementation of this part of the EAU guidelines.

The disease management in the Nordic countries is less uniform compared to the diagnostic practice for UTUC and treatment of bladder cancer [10]. EAU guidelines recommend endoscopic treatment for low-risk tumors and ureteral resection for low-risk tumors not eligible for endoscopic treatment, as well as high-risk tumors limited to distal ureter and other high-risk tumors when preservation of renal function is imperative. However, the majority of participating centers treat ureteral tumors by segmental resection, however most centers do not select eligible patients as recommended by EAU guidelines. This finding could in part be explained by the low incidence of ureteral tumors, combined with the lack of high evidence studies on the subject [12–14].

The survey also addressed if participating centers performed LND, although we did not ask to distinguish between low- and high-risk tumors, therefore wording of this question is not specific enough to determine adherence of guidelines on this subject. On the other hand, we asked participants if they carry out template-based LND concomitant to NU in all cases or only when lymph node metastasis was suspected clinically (cN+).

The EAU guidelines recommend LND offered to all patients who are scheduled for NU for high-risk non-metastatic UTUC, but the authors draw attention to the current lack of evidence of benefit in case of < T2 UTUC [8, 15, 16]. In proposed flowchart for disease management, high-risk UTUC is recommended treated by NU “+/- LND”, and not “+ LND”. To our knowledge, no randomized studies exists and prior studies have produced conflicting results [8, 15–21].

Internationally there is no consensus on indication for LND, which leave room for individual interpretation.

The lack of adherence to LND in approximately two-third Nordic centers is comparable to findings by others [16–18], and may be attributable to the absence of clear indications for LND. On the other hand, an ongoing prospective LND-trial in seven Nordic hospitals might have contributed to that some centers reported use of LND concomitant to NU. Instead, we investigate if participants use cN+, as a preoperative surrogate for tumor stage > pT1 as criteria for doing LND, as this practice seems to be supported by node positive disease as a trigger for adjuvant systemic therapies for UTUC [22]. Studies on usage of topical agents in the upper urinary tract for treatment of CIS and small low-grade tumors is mentioned in the EAU guidelines. The guideline members state that further research is needed and from existing evidence, treatment is with questionable efficacy. The same is true for neoadjuvant chemotherapy, but use adjuvant chemotherapy is strongly recommend to for high-risk non-metastatic UTUC [2]. Our study shows low adherence to this latter recommendation as the majority administer adjuvant chemotherapy only in selected cases. However, one might suspect that at least centers who use adjuvant chemotherapy “in selected cases” routinely do consider giving adjuvant chemotherapy but refrain due to postoperative carboplatin-ineligibility related to renal insufficiency. Alternatively, it is possible that some participants by “selected cases” mean high-risk UTUC as recommended by guidelines. Due to non-specific wording of the question, this cannot be further examined from current data.

Among surveyed Nordic centers 43% (17/47) performs > 10 NU/year. This might imply a higher degree of centralization of complex surgical procedures in the Nordic countries compared to in the US, as Sui et al. found that hospitals that performed > 6 NU/year represented only 9% of all included hospitals, while majority (71%) performed less than 3 NU/year and with an overall range of surgical volume, extending from 0.8 to 30.1 NU/year [23].

It is well established that hospital volume represents an important outcome determinant for several surgical treatments including urological [23–29]. Other studies claim that observed benefit on outcome is largely mediated by surgeon volume [26] and high procedure-specific volume [23, 30].

Sui et al. and Tinay et al. have shown that patients treated at centers performing > 6 NU/year who had a shorter length of stay (LOS) [31] and were more likely to receive intravesical chemotherapy perioperative [23]. However, the observed difference between the two groups was small.

As seen in Fig. 4b, low volume centers were less likely to administer intravesical chemotherapy in our survey, but the association was tested and found independent. Likewise, our data shows no association between surgical volume and estimated LOS in the survey. The different finding from our study compared to a US population-based study [31] could be explained by an inadequate number of participants in the current survey or the fact that LOS is estimated in this survey.

Only 64% based their answers on registries or local data collection.

### Limitations

As for all survey studies, there is a risk of inconsistencies between the responses and the actual practice patterns, recall/-reporting bias due to the wording of questions and the questions used in the survey were not validated. The selection of invited participants was made solely based on the knowledge by the representatives of NUCG. Additionally, it is not possible to conclude from which time period responses referred to or that the validity of the responses can be questioned, as one third of responding participants state that they do not register operative data on UTUC. As inclusion criteria centers were believed to perform NU, data might be missing on organ sparing procedures if some Nordic centers perform these treatments but not NU. For parameters like surgical volume and LOS, shorter numerical intervals would have made the data more comparable to other studies and might have enlightened a hospital-volume-outcome relationship.

As no power and sample size estimations was done, the interpretations of statistical analyses should be done with caution. Despite these limitations, we believe this study adds information and basis for further studies on treatment of UTUC in the Nordic countries.

## Conclusion

Adherence to EAU guidelines was high on diagnostic practice, but adhered to at a lesser degree when it comes to disease management. This dataset suggests that the Nordic countries have a good foundation for a common prospective database, as the low incidence and many diverse diagnostic and treatment modalities in UTUC necessitates multinational collaboration to investigate clinically meaningful outcomes in subgroups of patients with UTUC.

### Electronic supplementary material

Below is the link to the electronic supplementary material.

Supplementary Material 1

Supplementary Material 2

Supplementary Material 3

Supplementary Material 4

## Data Availability

The datasets used and analyzed during the current study are available from the corresponding author on request.
