# Supplementary material for: Perioperative management of upper tract urothelial carcinoma in the Nordic countries
Source: BMC Urol. 2024 Jun 25;24:132. doi: 10.1186/s12894-024-01515-7 (PMC11197368; doi:10.1186/s12894-024-01515-7)
Supplement: Supplementary file 1 — Supplementary Material 1 [file 12894_2024_1515_MOESM1_ESM.docx]

**Supplemental document 1** Questionary

**Name of department?**

**Country?**

**How many nephroureterectomies do your department do per year?**

< 10 - < 50 - 50-75 - 76-100 - >101

**Do your department also do cystectomies?**

Yes no

**Catchment population for UTUC patients?**

number - don’t know

**Do you receive UTUC patients from other departments?**

Yes - no - only for nefroureterectomy

**Do you routinely offer DNA sequencing for patients at high risk for Lynch syndrome/HNPCC?**

yes – no

**Do you formally do follow up on LYNCH patients?**

Yes- no

**Are any of the following modalities used as primary routine examination for TNM classification?**

CT urography - PET/CT scan i all cases - PET/CT scan in selected cases - MR- urography in all cases - MR- urography in selected cases - only perioperative retrograde ureteropyelography and/or selective cytology

**Do you do preoperative renography?**

Yes - no - in most cases

**Do you use diagnostic ureteroscopy preoperative?**

Always – only if imaging and cytology are not sufficient for the diagnosis – other criteria: describe

**Witch team of surgeons perform the ureteroscopy?**

Bladder cancer team – kidney cancer team – stone team - all teams – other: describe

**Do you collect peroperative urinary cytology of the renal cavities and ureteral**

**lumina during ureteroscopy?**

yes, always – no – in special cases: describe

**Do you biopsy tumors before treatment?**

Yes- no – in most cases – in few selected cases: describe

**Do you use preoperative risk factors according to EAU guidelines to stratify cases in high and low risk for therapeutic guidance?**

Yes – no – no, we stratify according to suspected TMN-stage non-invasive/invasive preoperatively – other: describe

**Who participates in your multidisciplinary preoperative cancer conferences?**

Urologist – Radiologist - Pathologist - Oncologist - Clinical physiologist/nuclear medicine - Secretary- Nurse – others?

**Do you use formal exclusion criteria as when selecting patients for radical nefroureterectomy?:**

Chronological age – kidney function – performance status – prior surgery - none of the above

**Do you use preoperative oral bowel preparation?**

never – only before nephroureterectomy – before endoscopic ablation and nephroureterectomy

**Do you use preoperative oral glucose load?**

never – only before nephroureterectomy – before endoscopic ablation and nephroureterectomy

**Do you use preoperative steroid as nausea prophylactic as a routine?**

never – only before nephroureterectomy – before endoscopic ablation and nephroureterectomy

**Do you give preoperative anticoagulation with i.e. LWH**

never – only before nephroureterectomy – before endoscopic ablation and nephroureterectomy

**Do you use an arterial cannula for invasive blood pressure during surgery?**

never – only before nephroureterectomy – before endoscopic ablation and nephroureterectomy

**Do you use a central venous line during surgery?**

never – only before nephroureterectomy – before endoscopic ablation and nephroureterectomy

**Do you ever treat low risk cancers by endoscopic ablation?**

If yes, is there formal criteria for which patients you offer the treatment?

**Witch team of surgeons perform the endoscopic ablation?**

Bladder cancer team – kidney cancer team – stone team - all teams – other : describe

**Do you offer kidney-sparing surgery to patients with low risk / non-invasive carcinoma?**

Never – Always – Only in cases of multi focal or “high burden” tumor - only in cases with serious renal insufficiency or having a solitary kidney – only in cases of fragility/comorbidity

**Do you ever use retrograde/antegrade instillation of BCG or mitomycin C in the upper urinary tract via JJ-stents/percutaneous nephrostomy?**

If yes, in which cases?

**Do you ever perform ureteric segmental resection as treatment for ureteral tumors?**

If yes, in which cases?

**Do you treat patients with high-risk tumors, according to EAU-guidelines, regardless of tumor location by radical nephroureterectomy?**

Yes- no

**Is the operation radical nephroureterctomy always including bladder cuff excision?**

yes – other: describe

**What number of surgeons are performing radical nephroureterectomy in your department?**

1 – (2-3) – (4-5) - (6-10) – (>10)

**Witch team of surgeons perform radical nephroureterectomy?**

Bladder cancer team – kidney cancer team - all teams – other: describe

**Method of radical nephroureterectomy?**

Open – laparoscopic - Robotic - Other

**What percentage of your radical nephroureterectomies are done**:

robotic - open – lap%

**Do you perform** **a template-based lymph node dissection during radical nephroureterectomy in case of cN0?**

Yes no – only in only in the case of:

**Do you perform** **a template-based lymph node dissection during radical nephroureterectomy in case of cN+?**

Yes no – only in only in the case of:

**Do you have biobank for upper urothelial carcinomas?**

Yes - No

**Do you use neo-adjuvant chemotherapy?**

Yes -no- in selected cases

if yes, then which regimen is used?

**Do you use adjuvant chemotherapy?**

Yes – no – in selected cases

if yes, then which regimen is used?

**Do you administer a single post-operative dose of intravesical chemotherapy after surgery?**

Yes, on POD: - no -in most cases

**If you use intravesical chemotherapy, how du you limit the chance of extravasation?**

By preoperatively testing the bladder with water – postoperative cystography – none of the above

**Do you use postoperative gastric tube?**

On which day do you remove the postoperative gastric tube, if used?

**Do you use abdominal drain?**

No- Yes, we remove the drain on POD: - in selected cases, we remove the drain on POD:

**Do you as your routine use perioperative antibiotics?**

No never - Yes- single dose perioperative - Yes- for 1 day - Yes-for 2 days - Yes for 3 days

**Which antibiotics are used?**

**Do you use postoperative nausea treatment.**

No - Yes formal - yes on demand

**Do you use postoperative laxatives?**

No – yes, on demand - yes formal

**On which day are the patient allowed to eat?**

Day 1 - When he/she wishes – after flatus - When he/she has bowel function – other: describe

**Do you use compression stockings?**

Yes - No

**As antithrombotic prophylactic we use?**

none - LWH until dismission- other duration: describe - Other type of anticoagulation

**Do you have a formal plan for mobilization?**

No - Yes from day 0 -Yes from day 1 - Yes when the patient is ready to mobilize

**Do you have standard nursing care plans?**

**Do you have formalized discharge criteria?**

No – If yes, then please describe them here:

**Do you register data on operative treatment of UTUC?**

No- if yes then how?

**Length of stay (median)**

0-1 day - < 2 days - < 3 days – 3 days or more – Don’t know

**What is the regime for follow-up after nephroureterectomy for low risk / pTa tumors at your institution?**

No control - cystoscopy at 3 + 9 months, then yearly, for five years - other: please describe

**In low risk / Ta tumors do you do urography after NU as follow up on contralateral kidney?**

Yes - no - in selected cases

**What is the regime for follow-up after nephroureterectomy for ≥T1 tumors at your institution?**

No control - cystoscopy + urinary cytology at every 3 months for 2 years there after every 6’th month for the next 3 years and then yearly – cystoscopy by the 4-8-12 month regimen for 5 years - individually - other: please describe.

**Do you register patients treated surgically for UTUC in a formalized prospective database?**

Yes - If no, please describe what your answers are based on
