# Supplementary figures and images for: Perioperative management of upper tract urothelial carcinoma in the Nordic countries
Source: BMC Urol. 2024 Jun 25;24:132. doi: 10.1186/s12894-024-01515-7 (PMC11197368; doi:10.1186/s12894-024-01515-7)

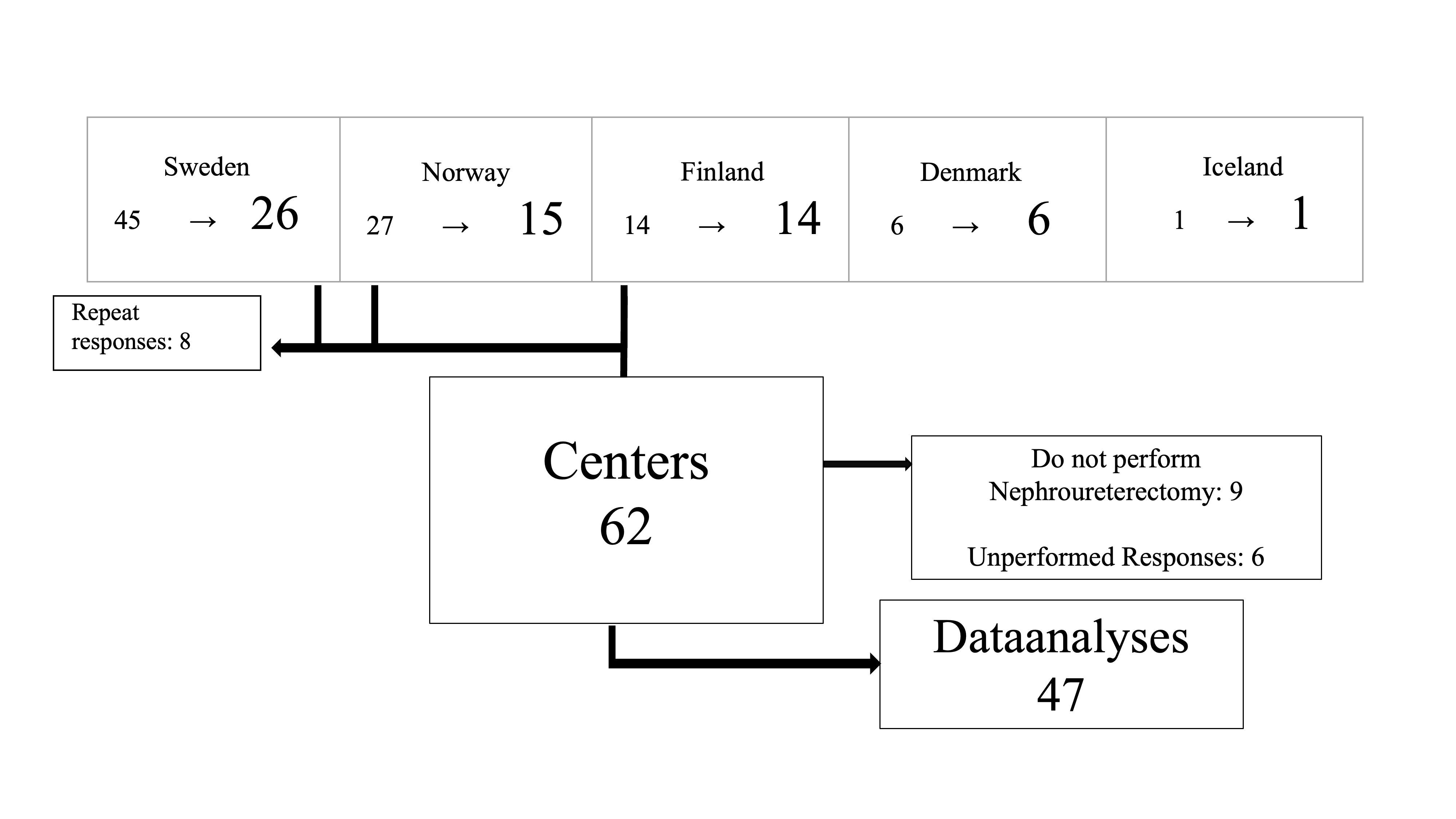

Supplement: Supplementary file 2 — Supplementary Material 2 [file 12894_2024_1515_MOESM2_ESM.tiff]

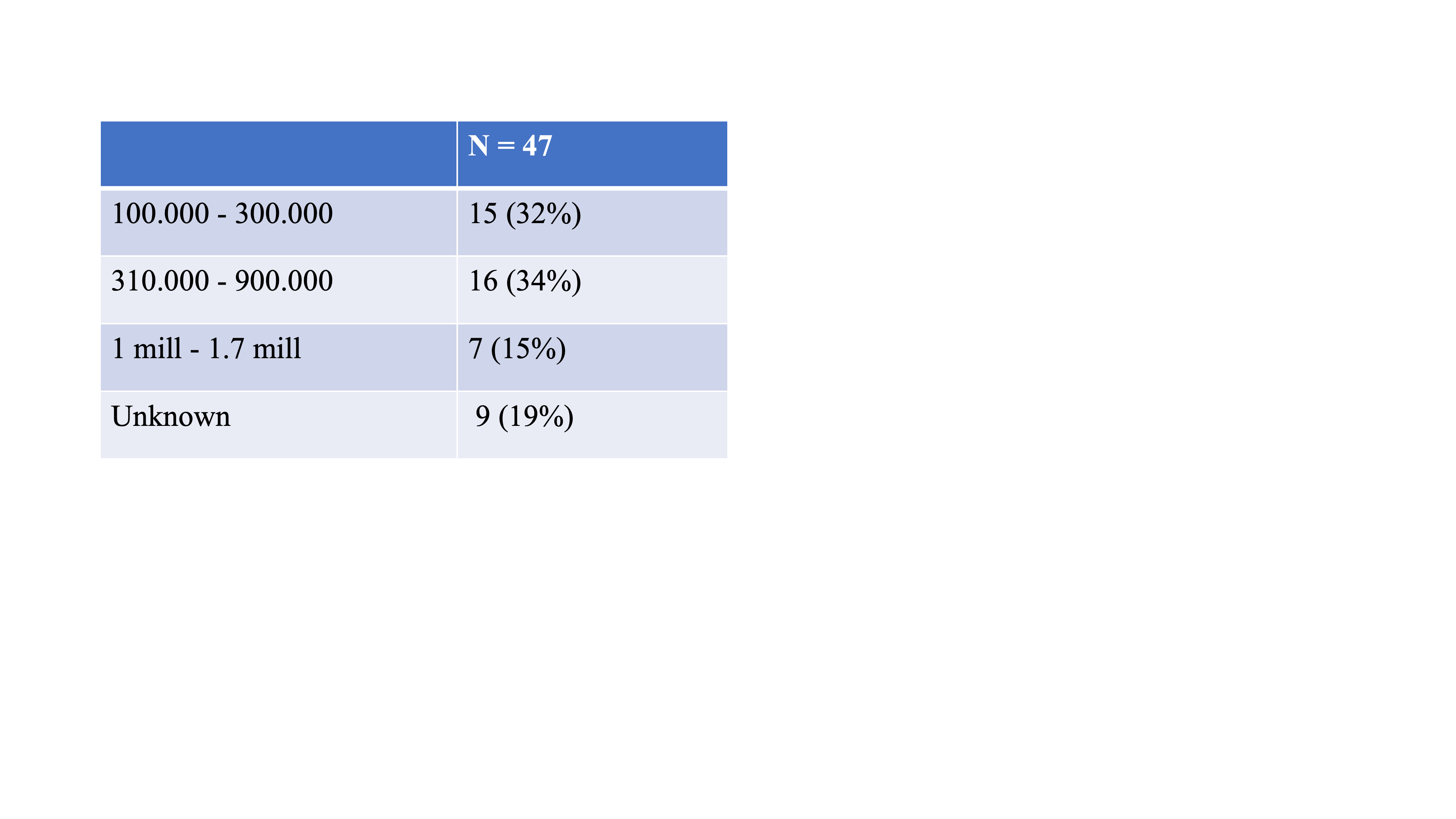

Supplement: Supplementary file 3 — Supplementary Material 3 [file 12894_2024_1515_MOESM3_ESM.tiff]

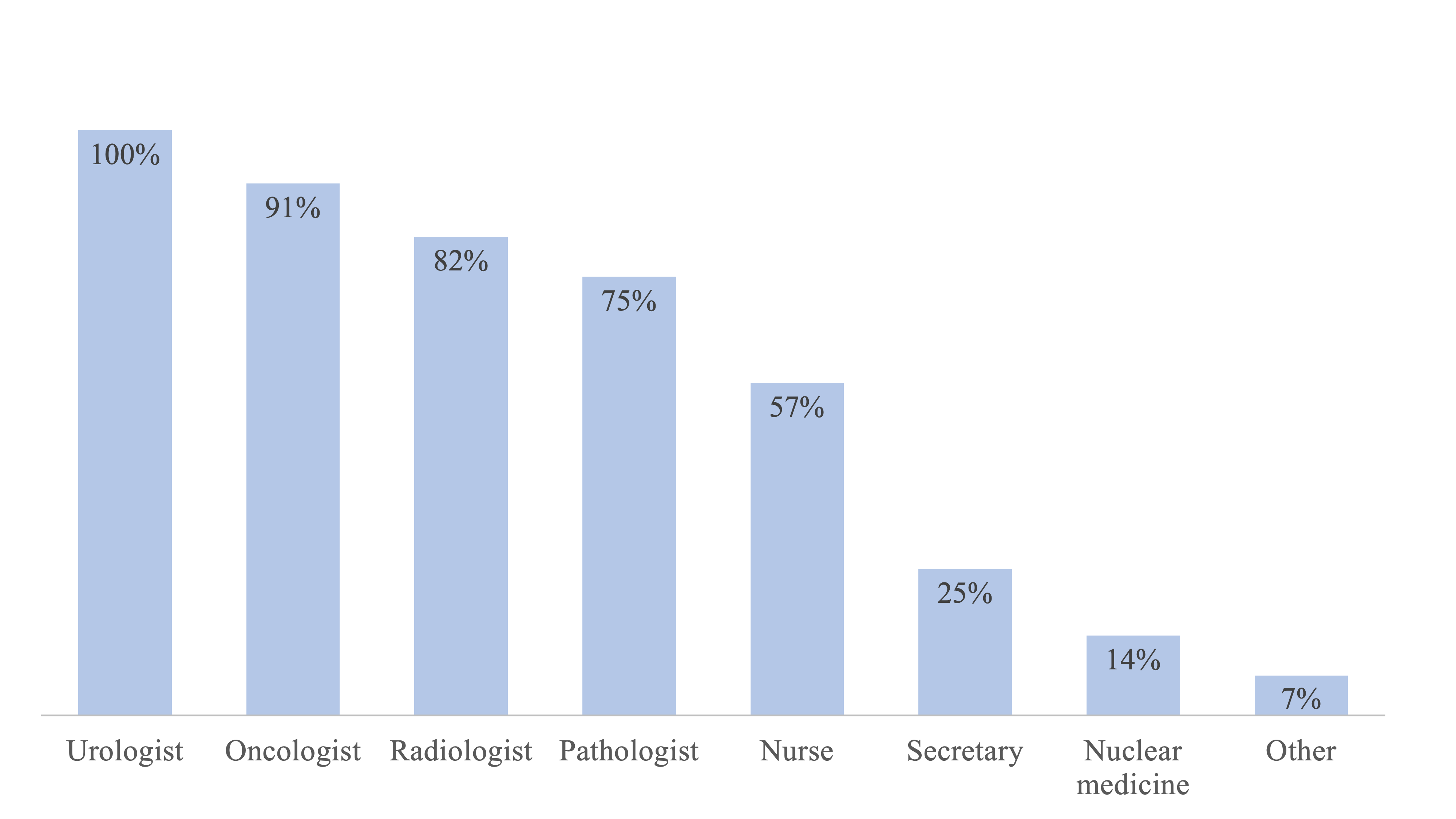

Supplement: Supplementary file 4 — Supplementary Material 4 [file 12894_2024_1515_MOESM4_ESM.tiff]
